# Characterization of Soil Bacteria with Potential to Degrade Benzoate and Antagonistic to Fungal and Bacterial Phytopathogens

**DOI:** 10.3390/microorganisms9040755

**Published:** 2021-04-03

**Authors:** Tatiana Z. Esikova, Tatiana O. Anokhina, Tatiana N. Abashina, Nataliya E. Suzina, Inna P. Solyanikova

**Affiliations:** 1 Laboratory of Plasmid Biology, Institute of Biochemistry and Physiology of Microorganisms, Pushchino Scientific Center for Biological Research of the Russian Academy of Sciences (PSCBR RAS), 142290 Pushchino, Russia; das3534@rambler.ru (T.Z.E.); to_anohina@rambler.ru (T.O.A.); 2 Laboratory of Cytology of Microorganisms, Institute of Biochemistry and Physiology of Microorganisms, Pushchino Scientific Center for Biological Research of the Russian Academy of Sciences (PSCBR RAS), 142290 Pushchino, Russia; tnabashina@gmail.com (T.N.A.); suzina_nataliya@rambler.ru (N.E.S.); 3 Laboratory of Microbial Enzymology, Institute of Biochemistry and Physiology of Microorganisms, Pushchino Scientific Center for Biological Research of the Russian Academy of Sciences (PSCBR RAS), 142290 Pushchino, Russia; 4 Regional Microbiological Center, Belgorod National Research University, 308015 Belgorod, Russia

**Keywords:** xenobiotics, degradation, soils, bioremediation, phytopathogens, plant protection

## Abstract

The intensive development of agriculture leads to the depletion of land and a decrease in crop yields and in plant resistances to diseases. A large number of fertilizers and pesticides are currently used to solve these problems. Chemicals can enter the soil and penetrate into the groundwater and agricultural plants. Therefore, the primary task is to intensify agricultural production without causing additional damage to the environment. This problem can be partially solved using microorganisms with target properties. Microorganisms that combine several useful traits are especially valuable. The aim of this work was to search for new microbial strains, which are characterized by the ability to increase the bioavailability of nutrients, phytostimulation, the antifungal effect and the decomposition of some xenobiotics. A few isolated strains of the genera *Bacillus* and *Pseudomonas* were characterized by high activity against fungal phytopathogens. One of the bacterial strains identified as *Priestia* *aryabhattai* on the basis of the 16S rRNA gene sequence was characterized by an unusual cellular morphology and development cycle, significantly different from all previously described bacteria of this genus. All isolated bacteria are capable of benzoate degradation as a sign of the ability to degrade aromatic compounds. Isolated strains were shown to be prospective agents in biotechnologies.

## 1. Introduction

The continuously increasing level of environmental pollution leads to the need for the development of biotechnologies for environment remediation. Soil is an extremely complex habitat, rich in microorganisms and characterized by a high diversity of microbial communities. The number of microorganisms is several billion cells per gram of soil, and the biodiversity reaches hundreds of thousands of species of bacteria and archaea [1]. 

The rhizosphere is the most microorganism-rich part of the soil due to the mutual positive influence of plants and microorganisms on each other.

Soil is not only a depository of biological diversity but also a kind of biochemical reactor, since microorganisms constantly carry out many enzymatic reactions/processes, including the degradation of xenobiotics and pollutants of natural origin [2,3,4,5]. The microbial community is the most important ecological indicator of “soil health”, which reflects the state of the soil biocenosis and its response to various influences, including pollution by toxic substances. Soils of agricultural importance are subjected to colossal anthropogenic impact, which leads to a change in their mineral composition, a decrease in the content of soil organic matter, the accumulation of pesticides, the spread of pesticide-resistant phytopathogenic microorganisms and, ultimately, to the depletion of the species and numerical composition of agrobiocenoses [6].

Among the research fields of soil microbiology, it is necessary to note such areas as the assessment of the general state of microbial systems, seasonal fluctuations in the biomass of the microbiocenosis and the spread of microorganisms of various taxonomic groups in soils of different types [7], as well as the study of the effect of the anthropogenic load on the change in abundance and the diversity of soil microorganisms [8]. Special attention is paid to the study of rhizosphere microorganisms, including bacteria that stimulate plant growth (plant growth-promoting rhizosphere bacteria) [9,10]. In this case, not only microbial diversity is investigated, but the features of the interactions of microorganisms with the host plant are also revealed. The mechanisms of the positive effect of bacteria on plants can be roughly divided into two types: direct stimulation of plant growth through the synthesis of phytohormones and improvement of their mineral nutrition and indirect stimulation of plant growth by inhibiting the growth of soil phytopathogenic fungi and bacteria [11].

In recent decades, biological preparations based on a variety of microorganisms and their metabolites are increasingly used to protect plants from pathogens [12,13]. The active agents of biopreparations are components of natural biocenoses, which explains their safety for the environment. The positive aspects of using microbiological preparations are their environmental friendliness, including a decrease in the chemical load on agroecosystems, low cost, the possibility of rotating them with chemical plant protection agents and a fairly high biological effectiveness of action. The use of biological products increases the yield of agricultural crops by 10–40% (depending on the type of plants) and improves the quality of products and their nutritional and feed values. In the Russian market, there are a number of such well-proven microbiological formulations, including inoculants for legumes (Rizotorfin); biofungicides (Flavobacterin, Rizoplan, Gamair and Glyocladin) and growth stimulants (Mizorin, Agrophil and BisolbiMix) [14,15].

Currently, the urgent tasks for increasing the productivity of agricultural crops are the isolation of new effective strains of bacteria to protect and stimulate plant growth and the study of their influence on plant development in various conditions, including in areas contaminated with toxicants, including pesticides. The aim of this work was to search for new microbial strains that are applicable for agricultural production and/or are able to degrade any pollutants.

## 2. Material and Methods

### 2.1. Bacterial Strains and Cultivation Conditions

Bacterial strains were isolated from the chernozem soil of the Belgorod Region, Russia, collected in March 2020 (GPS 50.558336,36.399521). These soils are used as agricultural soils with a crop rotation. To isolate strains, the selected samples (1.0 g) were resuspended in a mineral medium of the following composition (g L^−1^): Na_2_HPO_4_, 0.73; KH_2_PO_4_, 0.35; MgSO_4_ × 7H_2_O, 0.1; NaHCO_3_, 0.25; MnSO_4_ × 5H_2_O, 0.002; NH_4_NO_3_, 0.75 and FeSO_4_ × 7H_2_O, 0.02 (Reakhim, Poccия), pH 7.2. Soil suspension were diluted to 10^−8^ and 100 µL of diluted 10^−6^–10^−8^ samples were plated on mineral agar medium with 0.2 g L^−1^ of benzoate Na as the sole sources of carbon and energy. Bacteria were grown at 28 °C. Grown colonies were picked up and transferred on sterile agar Luria-Bertani (LB) medium [16].

Strains of phytopathogenic fungi *Gaeumannomyces graminis* var. *tritici*, *Fusarium graminearum* and *Rhizoctonia solani* (anastomosis group 5) were used as test cultures to study the antagonistic activity of the isolates. For the cultivation of fungi, Kanner’s medium was used [17].

Phytopathogenic bacteria Pseudomonas savastanoi VKM B-1546, Pantoea agglomerans ATCC 27155^T^, Ralstonia sp. 1–7, Pectobacterium carotovorum subsp. carotovorum VKM B-1247^T^, Xanthomonas campestris VKM B-610, X. campestris VKM B-570, Agrobacterium tumefaciens GV3101(pMP90RK) [18], A. tumefaciens CBE21 [19], Clavibacter michiganensis subsp. michiganensis VKM Ac-1403 and Pectobacterium wasabiae B15 (obtained as Erwinia carotovora B15) [20] were used as test cultures to study the antibacterial activity of the isolates. The P. chlororaphis strain BS1393, which produces a number of antibiotic-active compounds, was used as a control [21]. In the study of antibacterial activity, a 4-fold diluted LB medium supplied with 1.2 g L^−1^ of dextrose was used.

### 2.2. Determination of the Antagonistic Activity of Soil Strains

To determine the antifungal activity, the soil strains were grown in liquid LB medium for 16–20 h. An aliquot of the 18-h culture (5 µL) was applied to the surface of Kanner’s agar medium and incubated at 24 °C for 2 days. Then, a segment of the fungus mycelium (8–10 mm in diameter) was placed in the center of a Petri dish. The fungus mycelium was preliminarily grown in Kanner’s medium at room temperature for 7 days. Petri dishes were incubated at room temperature for 7–10 days, and then, the inhibition zone size of mycelium was assessed. The size of the growth inhibition zone was measured from the edge of the bacterial colony.

To determine the antibacterial activity, 100 µL of the phytopathogenic test culture (density 1–3 × 10^8^ colony-forming units (CFU)/mL) was spread over the surface of the LB agar medium. Then, 10 μL of the culture of the isolates was applied from above and incubated at a temperature of 24 °C for 7 days. The diameter of the growth inhibition zone was assessed over 2–7 days, considering the fact that the size of the bacterial colony was no more than 10 mm.

### 2.3. 16S rRNA Gene Sequencing and Phylogenetic Analysis

Genomic DNA was isolated from cells using a Fungal/Bacterial DNA Kit (Zymo Research, Irvine, CA, USA) according to the manufacturer’s recommendation. The 16S rRNA gene was amplified by PCR using primers universal for 16S rRNA prokaryotes: 27f (5′-AGAGTTTGATCCTGGCTCAG-3′) and 1492r (5′-TACGGYTACCTTGTTACGACTT-3′) [22]. The amplified DNA was purified using the Zymoclean Gel DNA Recovery Kit (Zymo Research, Irvine, CA, USA). Sequencing of the PCR DNA fragments was performed by the Sanger method on an Applied Biosystems 3130 Genetic Analyzer automatic sequencer (Applied Biosystems, Foster City, CA, USA) [23].

Primary phylogenetic screening of the obtained sequences was performed using the BLAST program [24] in the EzBioCloud database [25]. For the phylogenetic analysis, 16S rRNA gene sequences were taken from the GenBank database [26]. The nucleotide sequences of the 16S rRNA gene obtained for the strains were manually aligned with the sequences of the reference strains of the nearest microorganisms. A phylogenetic tree was constructed using partial 16S rRNA gene sequences by the neighbor-joining method [27] with a bootstrap test of 1000 replicates, performed using MEGA 6.0. [28].

The nucleotide sequences of the fragments of the 16S rRNA genes with lengths of about 1400 bp were deposited in the GenBank database under accession numbers MW642238 (*Bacillus subtilis* 18), MW642243 (*B. subtilis* 27), MW642237 (*B. subtilis* 28), EF114313 (*Priestia aryabhattai* 25) and MW659070 (*Pseudomonas chlororaphis* 3).

### 2.4. Microscopy

Light microscopy under phase contrast was carried out under a Nikon Eclipse Ci microscope (Nikon, Minato, Japan) equipped with a Jenoptic ProgResSpeedXTcore5 camera (Jenoptic, Jena, Germany).

To perform electron microscopy of thin sections, the cell biomass was prefixed with 1.5% (*v*/*v*) glutaraldehyde solution in 0.05-M cacodylate buffer (pH 7.2) at 4 °C for 1 h. After three washings with the same buffer, the material was additionally fixed with 1% OsO_4_ in 0.05-M cacodylate buffer at 20 °C for 3 h. After dehydration, the material was embedded into the epoxy resin Epon 812. Ultrathin sections were made on an 8800 ULTROTOME III (LKB-Produkter, Stockholm, Sweden). The sections were mounted on copper grids covered with a Formvar film, contrasted with uranyl acetate (3% solution in 70% ethanol) for 30 min and then stained with lead citrate [29] at 20 °C for 4 to 5 min. The sections were examined in a JEM- 1200EX (JEOL, Tokyo, Japan) electron microscope at an 80-kV accelerating voltage.

### 2.5. Characterization of the Biochemical Properties of the Isolated Strains

To determine the spectrum of utilized substrates and enzyme activities of the isolates, we used the Analytical Profile Index (API) 20 E and CH 50 strips (bioMerieux, Marcy-l’Étoile, France) according to the manufacturer’s instructions, as well as colored Hiss medium with carbohydrates that contained the following (g L^−1^): peptone, 10.0; NaCl, 5.0; carbohydrate, 7.0 and 1.6% bromothymol blue solution, 1.0 mL.

### 2.6. Determination of the Degradative Potential of the Isolated Strains

The bacteria were tested for their ability to utilize various aromatic, aliphatic and chlorine-containing compounds that were added to the mineral medium as the sole source of carbon and energy. The substrates were used in concentrations: caprolactam −1.0 g L^−1^; salicylate, gentisate, protocatechoate, o-phthalate, phenol, benzoate, chlorobenzoates (2-, 3-, 4-chlorobenzoate) and 2,4-dichlorophenoxyacetic acid (2,4-D) −0.2 g L^−1^ and 2,4,6-trichlorophenol −0.1 g L^−1^. Volatile aromatic and aliphatic compounds (naphthalene, toluene, ethylbenzene, m-xylene, p-xylene, diesel fuel, octane, hexane and hexadecane) were applied to the lid of an inverted Petri dish.

Bacterial growth on glyphosate (0.5 g L^−1^) as the sole source of phosphorus was tested as described previously [30].

### 2.7. Statistical Data Processing

Mean values and standard deviations were calculated based on the data of three independent experiments using the Microsoft Excel 2007 program [31]. 

## 3. Results and Discussion

### 3.1. Antagonistic Activity

By the method of direct inoculation on agar medium with sodium benzoate as the sole growth substrate, 30 bacterial strains were isolated, differing in colony morphology.

Recently, for the development of biological products, preference has been given to strains that combine such useful properties as the biocontrol of phytopathogens and stimulation of plant growth, as well as the ability of the biodegradation of xenobiotics. For this reason, all isolates were tested for antagonistic activity against phytopathogenic fungi and bacteria, which are the most dangerous infecting agents of plant diseases.

**Figure 1:**
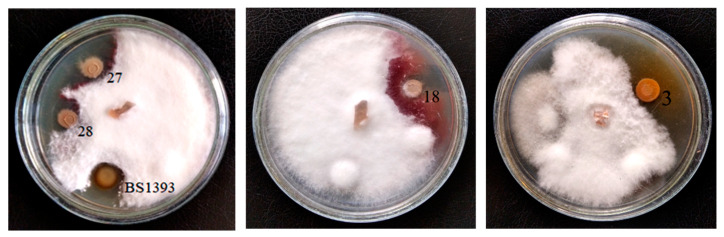
Antifungal activity of the soil strains 3, 18, 27 and 28 towards *Fusarium graminearum.*

Initially, the ability of soil strains to inhibit the growth of fungi *Gaeumannomyces graminis* var. *tritici*, *Fusarium graminearum* and *Rhizoctonia solani* was studied. Only four strains (designated as 3, 18, 27 and 28) exhibited pronounced antifungal activity. Of these isolates, three had the greatest activity against all used phytopathogenic fungi. The size of the growth inhibition zones of the test microorganisms was not less than in the control strain *P. chlororaphis* BS1393. Bacteria 18, 27 and 28 suppressed the growth of *F. graminearum* only (Table 1 and Figure 1).

**Figure 2:**
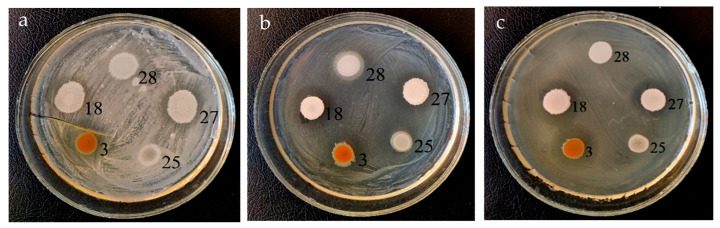
Antibacterial activity of soil strains 3, 18, 25, 27 and 28 towards *Ralstonia* sp. 1-7 (**a**), *Pectobacterium carotovorum* subsp. *carotovorum* VKM B-1247T (**b**) and *Pectobacterium wasabiae* B15 (**c**) after 7 days of cultivation.

Further, these strains (3, 18, 27 and 28) were studied for their ability to inhibit the growth of the phytopathogenic bacteria belonging to the genera *Pseudomonas*, *Pantoea*, *Ralstonia*, *Pectobacterium*, *Xanthomonas*, *Agrobacterium* and *Clavibacter*. Strain 3 exhibited the highest antibacterial activity and effectively suppressed nine out of 10 test bacteria (Table 2 and Figure 2). The diameter of the growth inhibition zones, as a rule, was larger than that of the control strain *P. chlororaphis* BS1393. A significant difference between strains 3 and 1393 was found in the suppression of phytopathogenic bacteria *Ralstonia* sp. 7-1, *X. campestris* B-610, *A. tumefaciens* CBE21 and *C. michiganensis* Ac-1403.

Representatives of the genera *Pseudomonas* and *Bacillus*, among which are many endophytic and rhizospheric species, are described [32,33]. These bacteria are characterized by the ability to directly or indirectly influence plant growth, including controlling the growth of phytopathogenic microorganisms [32]. It is known that some strains of fluorescent *Pseudomonas* bacteria can produce a wide range of antibiotic active metabolites that protect plants from various phytopathogenic microorganisms. An example of such antibiotics are phenazines, colored heterocyclic nitrogen-containing compounds produced almost exclusively by bacteria in the late exponential and stationary growth phase. The ability of *Pseudomonas* bacteria to control the growth of phytopathogens by the synthesizing of the phenazine or pyrrolnitrin antibiotics was the reason for the increased interest of researchers in this group of bacteria [34,35]. Strain 3, identified as *P. chlororaphis* (see Section 3.3. Strains identification), was characterized by a bright orange color of colonies, which may indicate to the production of 2-hydroxylated derivatives of phenazine-1-carboxylic acid (unpublished data). It was previously shown that 2-hydroxyphenazines exhibited strong bacteriostatic and fungistatic activity [36]. Probably, the synthesis of phenazine derivatives is the reason for the high antagonistic activity of this strain. Some strains of *Pseudomonas* were shown to have significant antagonistic effects against many different organisms, such as rootworms, *Fusarium oxysporum*, *F. graminearum*, *Gaeumannomyces graminis*, *Phytophthora capsici*, *Pythium ultimum* and *Sclerotinia* spp. [34,35,37].

Strains 18 and 27 were characterized by lower antibacterial activity and suppressed seven out of 10 phytopathogenic bacteria. Strain 28 inhibited the growth of only *A. tumefaciens* GV3101(pMP90RK) and *A. tumefaciens* CBE21. None of the strains inhibited the growth of *Pantoea agglomerans* ATCC 27155TM (formerly *Erwinia herbicola* ATCC 27155). *Bacillus subtilis* and *B. amyloliquefaciens* were among 133 bacterial strains from 11 composted aromatic plant wastes isolated for their ability to inhibit the growth of the mycelium of soil phytopathogenic fungi *Sclerotinia minor* and *Rhizoctonia solani* [38]. Gram-positive bacteria *Bacillus* spp. is also among the best-studied antagonist microorganisms for biological control in agriculture. *B. subtilis* ACB-83 produced two antibiotics, iturin and surfactin, and was successfully used to prevent and control citrus black spots caused by the fungus *Phyllosticta citricarpa* [39]. *Bacillus velezensis* 83 was isolated from the mango tree phyllosphere of orchards [9]. This strain can be listed as an example of the ability of bacilli to carry out a protective function, synthesizing compounds that can inhibit the growth of phytopathogens and acting as producers of compounds that stimulate plant growth and development. “In vivo assays *B. velezensis* 83 was shown to be able to control anthracnose (Kent mangoes) as efficiently as chemical treatment with Captan 50 PH™ or *Cupravit hidro*™. The inoculation of *B. velezensis* 83 to the roots of maize seedlings yielded an increase of 12% in height and 45% of root biomass, as compared with uninoculated seedlings” [9]. Thus, bacteria of the genera *Pseudomonas* and *Bacillus* isolated from soil are promising agents for use as an active component of microbiological formulations.

### 3.2. Microscopic Studies

**Figure 3:**
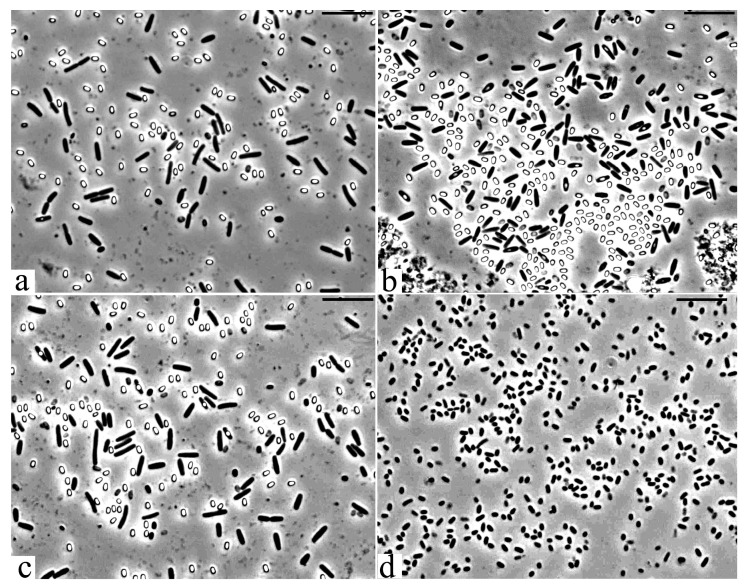
Cells of the studied strains in the exponential growth phase. (**a**) *Bacillus subtilis* 27, (**b**) *Bacillus subtilis* 28, (**c**) *Bacillus subtilis* 18 and (**d**) *Pseudomonas chlororaphis* 3. Light microscopy. Phase contrast. Bar—10 μm.

All 30 isolated cultures were microscopically examined for purity and morphological evaluation. Based on the results of tests for antifungal activity and microscopy, five cultures were selected for further work. The morphometric analysis of the phase contrast images of the cells of the studied bacterial strains made it possible to estimate their sizes and characterize a number of unique morphological features. The vegetative cells of studied bacterial strains 18, 27 and 28 are represented by large rods of regular shapes with the following sizes (Figure 3a–c): strain 27: 3.5–5 × 0.7–0.9 µm, strain 28: 2.5–3.5 × 0.9–1.0 µm and strain 18: 3.5–5 × 0.8–1.0 µm. The vegetative cells of bacteria 3 have characteristic sizes 1.2–1.8 × 0.7–0.9 µm (Figure 3d). In strain 25, the cells were characterized by an unusual shape and greatly varied in size (2.5–4-µm-long and 0.8–1-µm-wide), depending on the age of the culture. Strain 25 was selected for further work due to its unique morphological features.

**Figure 4:**
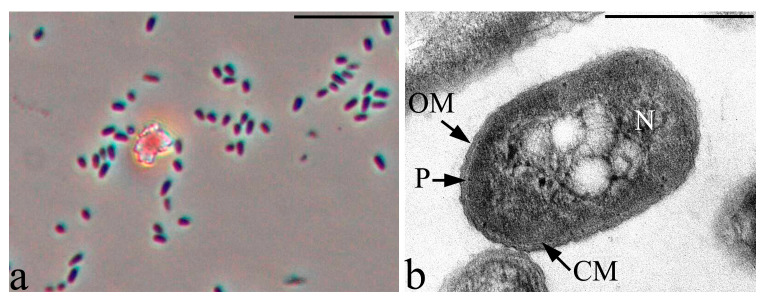
Phase contrast (**a**) and ultrathin section (**b**) of the strain 3 cells. Designations: OM—outer membrane, CM—cytoplasmic membrane, N—nucleoid and P—periplasm. Light microscopy. Phase contrast. Bar—10 µm. Transmission Electron Microscopy. Bar—0.5 µm.

Cells of strain 3 have a cell wall structure typical of Gram-negative bacteria with a characteristic outer membrane, a very thin layer of peptidoglycan (murein) and periplasm. In the nucleoid zone, the inclusion of regular spherical shapes is often present. In the cytoplasm, and often in the periplasmic space of cells, multiple small electron-dense inclusions are detected, apparently, as polyphosphates (Figure 4).

### 3.3. Strains Identification

**Figure 5:**
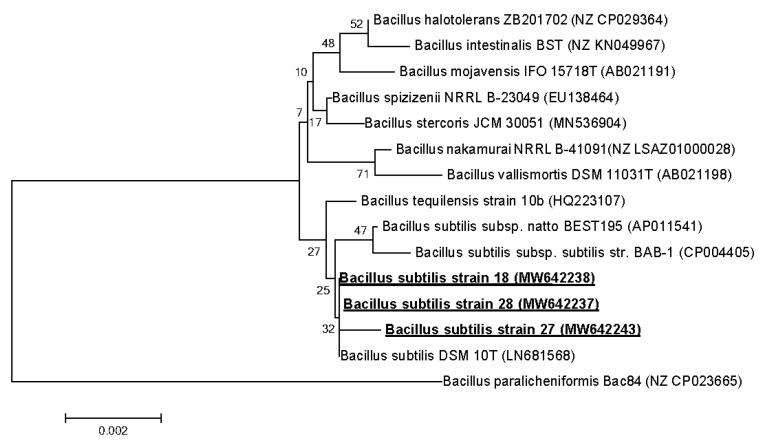
Phylogenetic position of strains 18, 27 and 28 based on the results of the comparative analysis of the nucleotide sequences of the 16S rRNA genes. The scale corresponds to 2 nucleotide substitutions per every 1000 nucleotides (evolutionary distance). The neighbor-joining method was used. The root was determined by including the sequence of *Bacillus paralicheniformis* Bac84 (NZ CP023665) as an external group.

The nucleotide sequences of the 16S rRNA gene (~1400 base pairs) for strains 18, 27 and 28 were determined. The sequences of the 16S rRNA gene in all strains differed in single-nucleotide substitutions, and, according to the results of the phylogenetic analysis, strains 18, 27 and 28 belong to the class Bacceliaceae, genus *Bacillus*. The strains showed the highest sequence similarity (99.4%) with the type of strain of this species *Bacillus subtilis* subsp. *subtilis* DSM 10^T^ (Figure 5).

The closeness of strains 18, 27 and 28 to each other and to the *Bacillus subtilis* species is also confirmed by biochemical characteristics (API tests CH 50 and 20 E) (Table 3 and Table 4). *Bacillus* spp. 18, 27 and 28 differed from each other and from the type *B. subtilis* DSM 10^T^ strain by no more than five out of 50 phenotypic characters, which indicates their closeness and the possibility of them belonging to this species. Nevertheless, since no DNA–DNA hybridization data were obtained, and the physiological and biochemical profiles are different, we are talking about three different strains of *Bacillus subtilis*. The sequences of the gene encoding 16S rRNA *B. subtilis* 18, 27 and 28 were registered in the GenBank data base under access numbers MW642238 (*B. subtilis* 18), MW642243 (*B. subtilis* 27) and MW642237 (*B. subtilis* 28).

**Figure 6:**
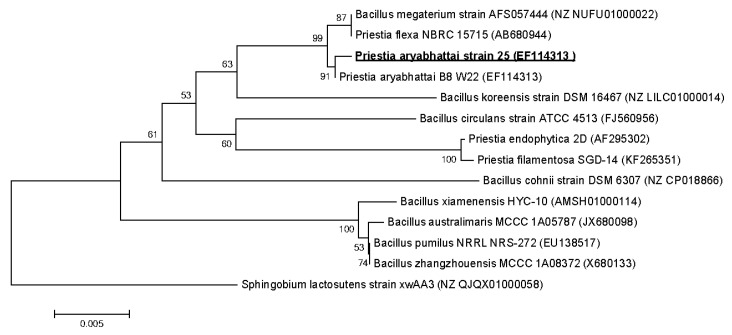
Phylogenetic position of strain *Priestia aryabhattai* 25 based on the results of the comparative analysis of the nucleotide sequences of the 16S rRNA genes. The scale corresponds to 5 nucleotide substitutions per every 1000 nucleotides (evolutionary distance). The neighbor-joining method was used. The root was determined by including the sequence of *Sphingobium lactosutens* strain xwAA3 (NZ QJQX01000058) as an external group.

The sequence analysis of the 16S rRNA gene of bacterial strain 25 showed that it belongs to the class *Bacceliaceae*, genus *Priestia* and has a high level of similarity (~99.6%) with the *Priestia aryabhattai* B8 W22. Sequence of the gene encoding 16S rRNA *P. aryabhattai* 25 was registered in the GenBank database under access number EF114313 (Figure 6).

**Figure 7:**
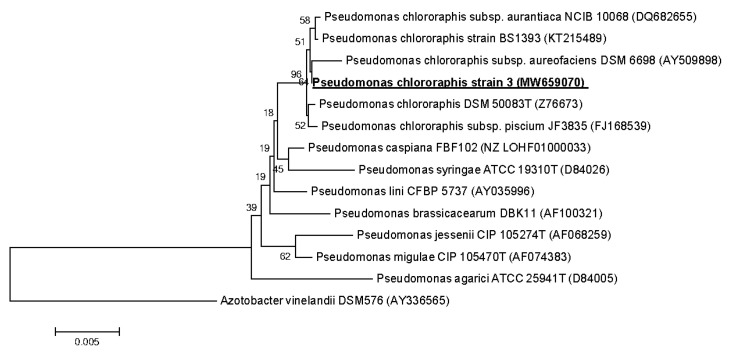
Phylogenetic position of strain *Pseudomonas chlororaphis* 3 based on the results of the comparative analysis of the nucleotide sequences of the 16S rRNA genes. The scale corresponds to 5 nucleotide substitutions per every 1000 nucleotides (evolutionary distance). The neighbor-joining method was used. The root was determined by including the sequence of *Azotobacter vinelandii* DSM576 (AY336565) as an external group.

A sequence analysis of the 16S rRNA gene of bacterial strain 3 showed that it belongs to the class Pseudomonadaceae, genus *Pseudomonas*, to the species *Pseudomonas chlororaphis* and has a high level of similarity (~99.6%) with the strain *Pseudomonas chlororaphis* DSM 50083^T^. The sequence of the gene encoding 16S rRNA *P. chlororaphis* 3 was registered in the GenBank database under access number MW659070 (Figure 7).

### 3.4. Biochemical Characteristics of Soil Strains

The determination of the biochemical characteristics of the cultures using the API 20 E and API 50 CH tests revealed the following features (Table 3 and Table 4). The biochemical profiles of strains 18, 27 and 28 were almost identical to each other and very close to the species *Bacillus subtilis* ATCC 6051^T^. All three strains were urease-negative. They showed the ability to liquefy gelatin and utilize citrates, as well as a wide range of carbon sources—namely, glycerol, L-arabinose, D-ribose, D-xylose, D-glucose, D-fructose, D-mannose, inositol, D-mannitol, D-sorbitol, salicin, D-cellobiose, D-maltose, D-melibiose, D-sucrose, D-trehalose and inulin. The studied strains cleaved potassium 2-ketogluconate and potassium 5-ketogluconate and also showed pronounced hydrolytic activity with respect to starch and glycogen, weak with respect to turanose.

*P. aryabhattai* strain 25, in contrast to strains 18, 27 and 28, showed the ability to utilize D-galactose and erythritol but not inositol and inulin. The strain had β-galactosidase and also possessed the ability to degrade N-acetylglucosamine, which indicates its potential antimicrobial activity.

*P. chlororhaphis* strain 3 utilized a wide range of organic substrates but did not hydrolyze starch, glycogen and xylitol and did not exhibit β-galactosidase and N-acetylglucoseamine enzyme activities. This strain was somewhat different in its biochemical characteristics from the *P. chlororaphis* strain ATCC 9446, which also belongs to the PGPR group of bacteria and is capable of synthesizing phenazine antibiotics [40]. For example, the latter strain fermented inositol, but not arabinose, in contrast to the strain we isolated.

### 3.5. Degradation Potential of Soil Strains

The strains *P. chlororaphis* 3; *B. subtilis* 18, 27 and 28 and *P. aryabhattai* 25 mentioned above were isolated on a mineral medium containing sodium benzoate as the sole source of carbon and energy. Benzoic acid is a naturally occurring aromatic compound widely distributed in the environment. When these strains were cultivated on a mineral medium with benzoate, no yellow color of the medium was observed in any case. This indicates that the isolated cultures implement the *ortho*- and not the *meta*- pathway of the cleavage of catechol [41]. This intermediate is the most frequently formed during microbial degradation of benzoate. Microorganisms capable of degrading benzoate, as a rule, convert some other aromatic compounds.

Our study showed that the selected bacteria more efficiently utilized monoaromatic carboxylic acids—salicylic (strains 18, 27 and 28); protocatechuic (strains 3 and 25) and *ortho*-phthalic (strains 27 and 28) (Table 5). The ability to grow on gentisic acid was observed only in the *P. aryabhattai* 25 strain. The strains also utilized such toxic organic compounds as *p*-xylene; hexadecane (*B. subtilis* 27 and 28); toluene (*P. aryabhattai* 25); diesel fuel (*B. subtilis* 27 and 28 and *P. aryabhattai* 25) and caprolactam (*B. subtilis* 18, 27 and 28 and *P. aryabhattai* 25). Bacterial growth was absent in the mineral medium supplemented with the chlorinated aromatic compounds 2-, 3-, 4-chlorobenzoate, 2,4-D, 2,4,6-trichlorophenol, naphthalene, phenol, ethylbenzene, *m*-xylene, octane and hexane.

It should be noted that only *P. chlororaphis* 3 grew on a medium containing glyphosate as the sole source of phosphorus, which is the basis of numerous herbicides registered in more than 120 countries under various trademarks. Owing to its application, residual amounts of glyphosate can persist for a long time in plants, soil and groundwater [42]. Therefore, the isolation of new strain-destructors, as well as increasing their activity, is an urgent task for the development of modern biotechnologies for the remediation of contaminated ecosystems.

The ability of soil microorganisms to catalyze the degradation reactions of various aromatic compounds can be considered as a basic ability of soil to self-purify and self-repair. As a rule, xenobiotic compounds enter the soil either as a result of an intense technogenic load on the soil or as a result of emergency situations. On the other hand, aromatic compounds are widespread in nature. Thus, benzoate is a common plant component. “Lignin is a structurally complex, heterogeneous, partly branched polymer synthesized from three main phenylpropane monolignols—coniferyl, sinapyl, and p-coumaryl alcohols. Softwood lignins are mainly composed of guaiacyl units originating from coniferyl alcohol, whereas hardwood lignin has both guaiacyl units and syringyl units originating from sinapyl alcohol” [43]. Thus, the presence of such naturally occurring molecules can be seen as a constant opportunity for bacteria to train their ability to degrade toxic or resistant compounds, which occurs during the coexistence of plants and bacteria.

### 3.6. Features of the Morphology of Priestia aryabhattai 25

**Figure 8:**
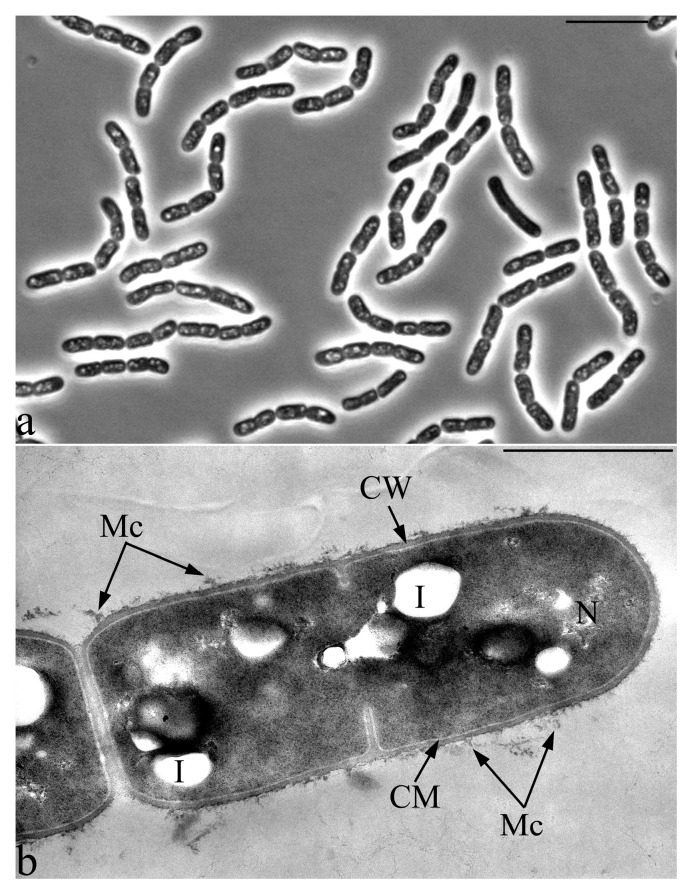
*Priestia aryabhattai* strain 25 in the phase of exponential growth (1 day) in the form of chains of rod-shaped cells with multiple intracytoplasmic inclusions. Designations: CM—cytoplasmic membrane, CW—cell wall, I—inclusion, N—nucleoid and Mc—microcapsule. (**a**) Light microscopy. Phase contrast. Bar—10 µm. (**b**) Transmission Electron Microscopy. Ultrathin section. Bar—1 µm.

**Figure 9:**
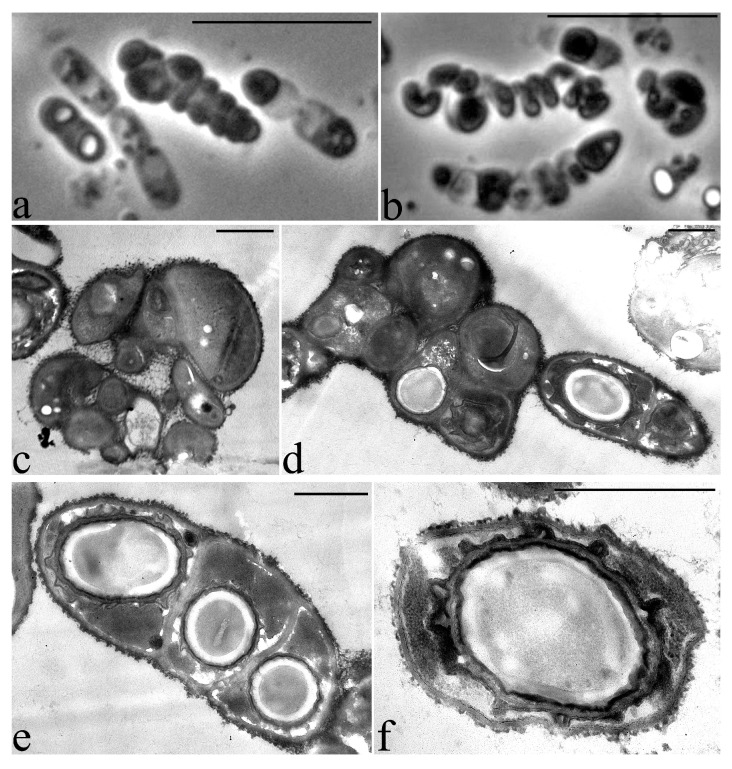
Phase contrast (**a**,**b**) and ultrathin sections (**c**–**f**) of the cleaving cells of *Priestia aryabhattai* strain 25 in a conglomerate (bunch). Some cells in the conglomerate are ultra-small (less than 300 nm in diameter). Light microscopy. Phase contrast. Bar—10 µm. Transmission Electron Microscopy. Bar—1 µm.

A number of unique morphological features of the bacterium *P. aryabhattai* 25 were identified by the morphometric analysis of the phase contrast images of the cells (Figure 8 and Figure 9). The bacterium *P. aryabhattai* 25 is distinguished by an unusual form of morphological rearrangements of cells in the cycle of culture development. Strain 25, in the process of exponential growth, forms chains of cells of irregular shapes, which, on the first day of growth on rich nutrient media, are filled with multiple refractory granules of unknown nature (Figure 8a).

On the second day of growth, the process of sporulation begins. During this phase of growth, some cells within the cell chain begin to divide in a fragmented manner, forming clusters of multiple small and ultrasmall irregular cell forms (Figure 9). Some of the ultrasmall cells in the clusters form spores (Figure 9d,e) and some form spirally twisted cords in which septa are subsequently formed, followed by fragmentation into ultra-small cell forms (Figure 9c,d). The formation of chaotically oriented cell walls in the cytoplasm can be seen on an ultrathin section of the growing strain 25 cell. There is an uneven division by the type of fragmentation, which is accompanied by the formation of small, up to 0.5 µm, and very small, around 0.3 µm, cells (Figure 9c,d).

The *P. aryabhattai* strain 25 is of great interest for understanding the processes of survival, the colonization of plant roots and the implementation of the interaction between the bacterium and the host plant. An analysis of the genomes of this bacterial species showed that most of them carry genes responsible for stimulating plant growth [44]. The peculiarity of strain 25, realized in the development cycle, to split into multiple ultra-small forms can contribute to the rapid and effective colonization of the rhizosphere of agricultural plants.

Recently, Muniraj et al. investigated the role of the bacterial isolate *Bacillus aryabhattai* TFG5 in the production of tyrosinase and its involvement in the production of humic substances from the waste of the coir pith [45]. The authors highlighted the role of the enzymes of this strain, tyrosinase and laccase, in the formation of humic substances in the soil. It is known that soil productivity is determined by the content of organic matter in it. Microorganisms play an important role in the restoration of soil fertility. Until recently, the main role in this process was assigned to fungi, due to the presence of a ligninolytic enzyme complex in them; thanks to which, fungi carry out the destruction of plant residues. The polymerization of phenolic molecules that originate from the degradation of lignin or the synthesis by microorganisms may lead to humic substances that can incorporate a variety of organic and inorganic molecules and elements [46]. Thus, the presence in soil bacteria, including *Bacillus aryabhattai*, of the activity of ligninolytic enzymes and tyrosinase may indicate an active role of bacteria of this species in the formation of soil fertility.

## 4. Conclusions

As a result of this study, a number of bacterial strains were isolated from the soil according to their ability to degrade benzoate. Some of these strains were proven to be antagonists of fungal and bacterial phytopathogens. Particularly noteworthy is the *P. chlororaphis* 3 strain, which was characterized by a pronounced ability to control the growth of the phytopathogens with comparable efficiency with the best bacterial strains used in biological products. In addition, the microscopic studies carried out allowed us to find the *P. aryabhattai* 25 strain, which was originally attributed to the genus *Bacillus*. However, both in the sequence of the 16S rRNA gene and in its biochemical properties and morphophysiological characteristics, it differs significantly from all known representatives of this genus, which made it possible to define it as *Prestia aryabhattai* strain 25. Thus, several new strains were isolated, which are of interest both as highly active agents controlling the growth of plant phytopathogens and as a representative of new groups of bacteria, the role of which for the environment still needs to be studied.

## Figures and Tables

**Table 1:** Antagonistic activity of the soil strains against phytopathogenic fungi.

Strain	Identification According 16S rRNA Gene Sequencing	Phytopathogen Suppression Zone, mm		
		*Fusarium* *graminearum*	*Rhizoctonia* *solani*	*Gaeumannomyces* *graminis* var. *tritici*
3	*Pseudomonas chlororaphis*	3 ± 1 ^1^	2 ± 1	2 ± 1
18	*Bacillus subtilis*	≤1	– ^2^	–
27	*Bacillus subtilis*	≤2	–	–
28	*Bacillus subtilis*	≤2	–	–
*Pseudomonas chlororaphis* BS1393		2 ± 1	3 ± 1	2 ± 1

^1^ The table shows the mean values ± standard deviations. The results were obtained from three independent experiments. ^2^ Zone of suppression of the growth of the test microorganism is absent.

**Table 2:** Antibacterial activity of the soil strains.

Test Bacteria	Diameter of the Suppression Zone, mm				
	*Pseudomonas* *chlororaphis* 3	*Bacillus* *subtilis* 18	*Bacillus* *subtilis* 27	*Bacillus* *subtilis* 28	*Pseudomonas* *chlororaphis* BS1393 (Control)
*Pseudomonas savastanoi* B-1546	16 ± 2 ^1^	14 ± 1	14 ± 1	–	14 ± 1
*Pantoea agglomerans* ATCC 27155 ^T^	– ^2^	–	–	–	–
*Ralstonia* sp. 7-1	26 ± 3	14 ± 1	14 ± 1	–	18 ± 3
*Pectobacterium carotovorum* B-1247 ^T^	16 ± 2	18 ± 2	18 ± 2	–	16 ± 2
*Pectobacterium wasabiae* B15	15 ± 2	18 ± 2	18 ± 2	–	12 ± 1
*Xanthomonas campestris* B-610	16 ± 1	–	–	–	12 ± 1
*Xanthomonas campestris* B-570	16 ± 2	14 ± 1	14 ± 1	–	13 ± 1
*Agrobacterium tumefaciens* GV3101(pMP90RK)	18 ± 2	12 ± 1	16 ± 2	16 ± 2	18 ± 2
*Agrobacterium tumefaciens* CBE21	26 ± 3	12 ± 1	12 ± 1	12 ± 1	18 ± 3
*Clavibacter michiganensis* Ac-1403	16 ± 1	–	–	–	13 ± 1

^1^ The table shows the mean values ± standard deviations. The results were obtained from three independent experiments. ^2^ Zone of suppression of the growth of the test bacteria is absent. ^T^ Type strain for this species.

**Table 3:** Range of the utilized substrates by soil strains.

Substrate	*Bacillus* *subtilis* 18	*Bacillus* *subtilis* 27	*Bacillus* *subtilis* 28	*Priestia* *aryabhattai* 25	*Pseudomonas* *chlororaphis* 3
Glycerol	+	+	+	+	+
Erythritol	-	+	-	+	-
D-arabinose	-	+	-	+	+
L- arabinose	+	+	+	+	+
D-ribose	+	+	+	+	+
D-xylose	+	+	+	+	+
L- xylose	-	-	-	-	-
D-adonitol	-	-	-	-	-
D-galactose	-	-	-	+	+
D-glucose	+	+	+	+	+
D-fructose	+	+	+	+	+
D-mannose	+	+	+	-	+
L-sorbose	-	+	-	-	-
L-rhamnose	-	-	-	-	+
Dulcitol	-	-	-	-	-
Inositol	+	+	+	-	+
D-mannitol	+	+	+	+	+
D-sorbitol	+	+	+	+	-
Methyl -αD- glucopyranoside	+	+	+	-	-
N-acetyl- glucosamine	-	-	-	+	-
Amygdalin	+	+	+	+	-
Arbutin	+	+	+	+	-
Esculin	+	+	+	+	+
Salicin	+	+	+	+	-
D-cellobiose	+	+	+	+	-
D-maltose	+	+	+	+	-
D-lactose	-	-	-	-	-
D-melibiose	+	+	+	+	+
D-saccharose	+	+	+	+	+
D-trehalose	+	+	+	+	+
Inulin	+	+	+	-	-
D-melezitose	-	-	-	+	-
D-raffinose	+	+	+	+	+
amidon (starch)	+	+	+	+	-
Glycogen	+	+	+	+	-
Xylitol	-	-	-	-	-
Gentibiose	+	-	-	+	+
D-turanose	+	+	+	+	-
D-lyxose	-	-	-	-	-
D-tagatose	-	-	-	-	-
D-fucose	-	-	-	-	+
L- fucose	-	-	-	-	-
D-arabitol	-	-	-	-	+
L- arabitol	-	-	-	-	-
Potassium gluconate	+	+	+	+	+
Potassium 2-ketogluconate	+	+	+	+	+
Potassium 5-ketogluconate	+	+	+	+	+

+ good growth, - no growth.

**Table 4:** Biochemical properties of the soil strains.

Reaction	*Bacillus* *subtilis* 18	*Bacillus* *subtilis* 27	*Bacillus* *subtilis* 28	*Priestia* *aryabhattai* 25	*Pseudomonas* *chlororaphis* 3
β-galactosidase	-	-	-	+	-
Arginine dihydrolase	-	+	-	-	+
Lysine decarboxylase	-	-	-	-	-
Ornithine decarboxylase	-	-	-	-	-
Citrate utilization	-	-	-	+	+
H_2_S production	-	-	-	-	-
Urease production	-	-	-	-	-
Tryptophane deaminase	-	-	-	-	+
Indole production	-	-	-	-	-
Voges Proskauer	+	+	+	-	-
Liquefaction of gelatin	+	+	+	+	+
Fermentation of glucose	+	+	+	+	+
Fermentation of mannitol	+	+	+	+	+
Fermentation of inositol	+	+	+	-	+
Fermentation of sorbitol	+	+	+	+	+
Fermentation of rhamnose	-	-	-	-	+
Fermentation of saccharose	+	+	+	+	+
Fermentation of melibiose	-	-	-	-	+
Fermentation of amygdalin	+	+	+	+	-
Fermentation of arabinose	+	+	+	+	+
NO_3_ reduction to NO_2_	-	-	-	-	-
NO_3_ reduction to N_2_	-	-	-	-	-

- absence of a property, + presence of a property.

**Table 5:** Growth of soil strains on toxic organic substrates.

Substrates	*Bacillus* *subtilis* 18	*Bacillus* *subtilis* 27	*Bacillus* *subtilis* 28	*Priestia* *aryabhattai* 25	*Pseudomonas* *chlororaphis* 3
Benzoate	+	+	+	+	+
Salicylate	+	+	+	-	-
Gentisate	-	-	-	+-	-
*o-*Phthalate	-	+-	+-	-	-
Protocatechuate	-	-	-	+	+
*p-*Xylene	-	+-	+-	-	-
Toluene	-	-	-	+-	-
Hexadecane	-	+-	+-	-	-
Diesel fuel	-	+-	+-	+-	-
Caprolactam	+	+	+	+-	-
Glyphosate	-	-	-	-	+

+ good growth, +- relatively slow growth and – no growth.
